# Inhibition of Lon protease by triterpenoids alters mitochondria and is associated to cell death in human cancer cells

**DOI:** 10.18632/oncotarget.4510

**Published:** 2015-07-15

**Authors:** Lara Gibellini, Marcello Pinti, Regina Bartolomeo, Sara De Biasi, Antonella Cormio, Clara Musicco, Gianluca Carnevale, Simone Pecorini, Milena Nasi, Anto De Pol, Andrea Cossarizza

**Affiliations:** ^1^ Department of Surgery, Medicine, Dentistry and Morphological Sciences, University of Modena and Reggio Emilia, Modena, Italy; ^2^ Department of Life Sciences, University of Modena and Reggio Emilia, Modena, Italy; ^3^ Department of Bioscience, Biotechnology and Biopharmaceutics, University of Bari, Bari, Italy; ^4^ CNR – Institute of Biomembranes and Bioenergetics, Bari, Italy

**Keywords:** mitochondria, Lon, CDDO, CDDO-Me

## Abstract

Mitochondrial Lon protease (Lon) regulates several mitochondrial functions, and is inhibited by the anticancer molecule triterpenoid 2-cyano-3, 12-dioxooleana-1,9(11)-dien-28-oic acid (CDDO), or by its C-28 methyl ester derivative (CDDO-Me). To analyze the mechanism of action of triterpenoids, we investigated intramitochondrial reactive oxygen species (ROS), mitochondrial membrane potential, mitochondrial mass, mitochondrial dynamics and morphology, and Lon proteolytic activity in RKO human colon cancer cells, in HepG2 hepatocarcinoma cells and in MCF7 breast carcinoma cells. We found that CDDO and CDDO-Me are potent stressors for mitochondria in cancer cells, rather than normal non-transformed cells. In particular, they: i) cause depolarization; ii) increase mitochondrial ROS, iii) alter mitochondrial morphology and proteins involved in mitochondrial dynamics; iv) affect the levels of Lon and those of aconitase and human transcription factor A, which are targets of Lon activity; v) increase level of protein carbonyls in mitochondria; vi) lead to intrinsic apoptosis. The overexpression of Lon can rescue cells from cell death, providing an additional evidence on the role of Lon in conditions of excessive stress load.

## INTRODUCTION

The neoplastic transformation of normal cells is a multistep process that is first initiated by diverse genetic alterations, then enhanced by several oncogenesis-associated cellular stresses, that can be observed in many cancer cells [1, 2]. Cancer cells develop in a microenvironment where stressors are greater than those present in normal cells, and for their survival have to activate several anti-stress pathways and defense mechanisms. Such pathways and mechanisms are sustained by a number of nononcogenic proteins, *i.e*. molecules which do not initiate tumorigenesis, but participate in the cell stress response, and heighten the survival and proliferation of cancer cells [3].

Among these proteins, a main role is played by Lon protease (Lon), a molecule highly conserved from bacteria to mammals, and that in *eukarya* is one of the quality control proteins within mitochondria [4]. It is encoded in the nucleus and it localizes to mitochondrial matrix, where controls mitochondrial function, especially under oxidative, hypoxic and metabolic-stress conditions. Lon recognizes damaged and oxidized proteins and mediates their proteolysis, acts as a chaperone, and is involved in mitochondrial DNA maintenance [5]. Aconitase and mitochondrial transcription factor A (TFAM) are known substrates of Lon proteolytic activity [6, 7].

Several lines of evidence support a role for Lon as a non-oncogenic protein essential for cancer survival. First, Lon expression increases in response to several stressors. In hypoxic cells, Lon is up-regulated and is responsible for degrading cytochrome *c* oxidase 4 subunit 1 (COX4-1) to optimize the efficiency of respiration [8]. Similarly, when cells are challenged with oxidative stress, Lon is involved in the degradation of misfolded, oxidized and carbonylated proteins, thereby preventing their accumulation [9, 10]. Second, Lon plays a key role in the remodeling of respiratory chain complexes during the metabolic reprogramming triggered in mitochondria in many cancer cells [11]. Knock-down of Lon activates the AMP-activated protein kinase (AMPK), which is a crucial regulator of the energy homeostasis under metabolic stress [12]. Third, Lon down-regulation in cancer cells results in disruption of mitochondrial function and structure, reduced proliferation, and increased apoptotic cell death [13]. Finally, Lon overexpression correlates with cancer cell aggressiveness, and indeed Lon is up-regulated in several cancer cells, including RKO colon carcinoma, HepG2 hepatocarcinoma, large cell lymphoma cell lines, Granta mantle cell lymphoma cell lines, and *ex vivo* specimens obtained from colon carcinoma and bladder cancer [3, 11, 13–16]. How Lon expression and functions are regulated is not well understood, but targeting its activity in cancer cells could represent a novel and valuable therapeutic strategy.

The synthetic triterpenoid 2-cyano-3, 12-dioxooleana-1,9(11)-dien-28-oic acid (CDDO), and its C-28 methyl ester derivative (CDDO-Me), are molecules with strong anti-inflammatory and anti-proliferative activity [17]. Several mechanisms have been proposed for their anticancer effect, such as: i) the formation of Michael adducts with reactive nucleophiles, including free thiols on target proteins, ii) the inhibition of mitogen activated protein kinase (MAPK) [18], iii) the induction of apoptosis through the mitochondrial pathway [19], and iv) the inhibition of Lon proteolytic activity [3]. Both CDDO and CDDO-Me interact with Lon and form covalent Lon-CDDO adducts that irreversibly inhibit Lon activity, thereby inducing mitochondrial protein aggregation [3]. We recently demonstrated that shRNA-mediated down-regulation of Lon in the human colon carcinoma cells RKO leads to impaired mitochondrial structure and function, causing apoptotic cell death *via* mitochondria [13]. Thus, we wondered whether treating different human cell lines, such as RKO, HepG2 and MCF7, compared to normal fibroblasts, with compounds that could have a potential interest for cancer treatment, *i.e*. CDDO or its methyl ester derivative, would provide the same effects found by shRNA knockdown of Lon. Thus, in human cancer cells, we characterized the effects of triterpenoids on mitochondria, paying a particular attention to membrane potential, mass, morphology, dynamics and ROS content.

## RESULTS

### CDDO and CDDO-Me decrease proliferation, induce apoptosis in cancer cells, and increase mitochondrial hydrogen peroxide and mitochondrial superoxide anion

**Figure 1:**
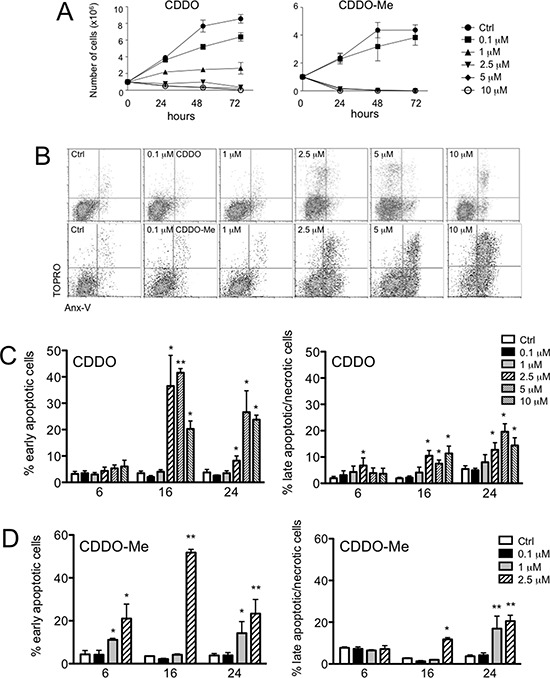

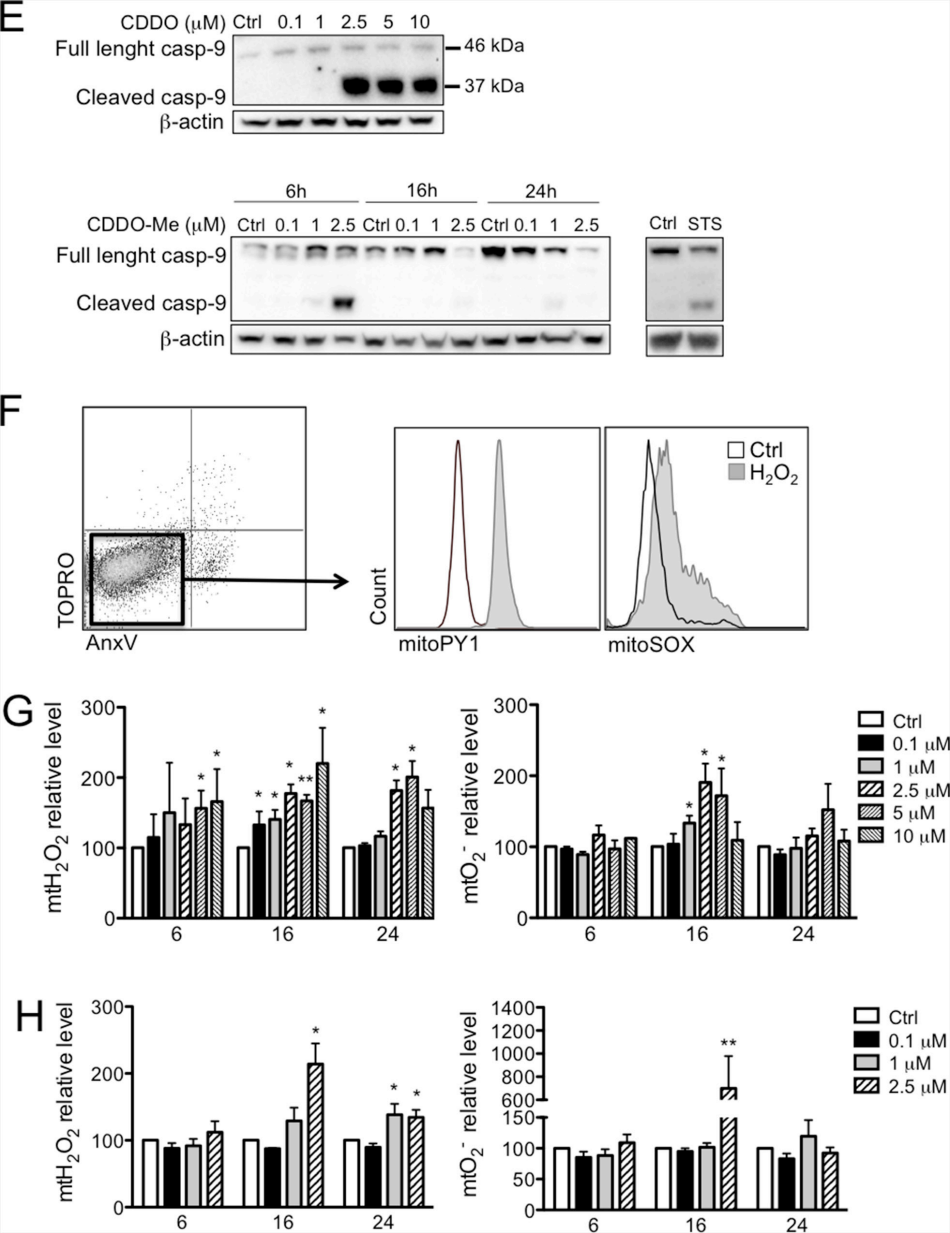
CDDO and CDDO-Me are anti-proliferative and induce apoptosis in RKO cells **A.** Growth curve of RKO cells incubated with the indicated concentrations of CDDO (left panel) or CDDO-Me (right panel) for up to 72 hours; DMSO-treated cells were considered as controls (Ctrl). **B.** Representative flow cytometric dot plots combining Annexin-V (AnxV) Alexa Fluor Pacific Blue and TO-PRO-3 iodide (TOPRO) staining in RKO cells treated with DMSO (Ctrl), or with increasing concentrations of CDDO or CDDO-Me for 24 hours. **C.** Percentage of early apoptotic cells (AnxV+/TOPRO- cells) and late apoptotic/necrotic cells (AnxV-/TOPRO+ and AnxV+/TOPRO+ cells) after treatment with the indicated concentrations of CDDO for up to 24 hours. Values represent the mean ± SD of five independent experiments, *P < 0.05 and **P < 0.01 *vs*. Ctrl. **D.** Percentage of early apoptotic cells and late apoptotic/necrotic cells after treatment with the indicated concentrations of CDDO-Me for up to 24 hours. Values represent the mean ± SD of five independent experiments, *P < 0.05 and **P < 0.01 *vs*. Ctrl. **E.** Western blot analyses showing caspase-9 activation in RKO cells after treatment with CDDO and CDDO-Me at the indicated concentrations. Cells treated with 1 μM staurosporine (STS) for 4 hours were used as positive controls for caspase-9 activation. **F.** Representative histograms showing MitoSOX Red Superoxide Indicator (mitoSOX) and Mitochondria Peroxy Yellow 1 (mitoPY1) to detect mitochondrial anion superoxide and mitochondrial hydrogen peroxide in living cells (*i.e*., those that were AnxV-/TOPRO- cells, indicated by the square). H_2_O_2_ was used as positive control. **G.** Quantification of mitochondrial hydrogen peroxide (mtH_2_O_2_) and mitochondrial anion superoxide (mtO_2_^−^) in RKO cells treated with CDDO up to 24 hours. Data are expressed as percentage of increase in median fluorescence intensity (MFI) and represent the mean ± SD of four independent experiments; *P < 0.05 and **P < 0.01 *vs.* Ctrl. **H.** Quantification of mtH_2_O_2_ and mtO_2_^−^ in RKO cells treated with CDDO-Me for up to 24 hours. Data are expressed as percentage of increase in MFI and represent the mean ± SD of four independent experiments; *P < 0.05 and **P < 0.01 *vs.* Ctrl.

We first analysed the effects of CDDO and CDDO-Me on cell growth by culturing RKO, HepG2 and MCF7 cells in the presence of increasing concentrations of these molecules for up to 72 hours. As shown in Figure 1A and in Supplementary Figure S1A, counting adherent cells revealed that a time- and concentration-dependent inhibition of cell proliferation was observed either for CDDO or for CDDO-Me, for RKO, HepG2 and MCF7 cells. However, CDDO-Me was much stronger than CDDO in inhibiting cell growth in all cell lines.

To investigate the inhibitory mechanisms induced by these drugs, we analysed their effects on apoptosis, and on mitochondrial functionality and morphology. First, we analysed simultaneously apoptosis and production of reactive oxygen species (ROS) at the single cell level by multicolour flow cytometry. Cells were first loaded with mitochondria peroxy yellow 1 (mitoPY1), which specifically detects mitochondrial hydrogen peroxide (mtH_2_O_2_), and then treated for up to 24 hours with CDDO or CDDO-Me. Supernatants were collected and cells stained with MitoSOX red superoxide indicator (MitoSOX), and with annexin-V (AnxV), and TO-PRO-3 iodide (TOPRO). Early apoptotic cells were those positive for AnxV and negative for TOPRO, whereas late apoptotic/necrotic cells were positive for TOPRO, independently on AnxV (Figure 1B). Also in this case, CDDO-Me was more potent than CDDO in causing cell death for RKO, HepG2 and MCF7 cells (Figure 1C and 1D, and Supplementary Figure S1B, and S1D): a slight increase in cell death was observed after 6 hour incubation with CDDO and 1–2.5 μM CDDO-Me. In RKO cells, after 16 and 24 hours of incubation, both CDDO and CDDO-Me led to a strong increase in the percentage of early apoptotic cells (Figure 1C and 1D, left panels). Concerning HepG2 cells, higher concentrations of CDDO and CDDO-Me were needed to induce apoptosis (Supplementary Figure S1B and S1C). We also investigated whether triterpenoids could affect viability of normal cells. Thus, we obtained primary cultures of human fibroblasts and we treated these cells with increasing concentrations of CDDO and CDDO-Me for 6, 16 and 24 hours. We found that CDDO and CDDO-Me did not cause apoptosis in human fibroblasts, except for the highest tested concentration of CDDO-Me, and only after 16 and 24 hours of incubation (Supplementary Figure S2A and S2B).

We next examined the effects of CDDO and CDDO-Me on the activation of caspase-9 and caspase-3, to understand if apoptotic cell death was mediated by the intrinsic pathway. As shown in Figure 1E, we were able to detect both the activation of caspase-9, evidenced by the presence of the band at 39 kDa, and the decreased expression of the full length protein (47 kDa), at least after 24 hours of treatment with CDDO-Me. The concentration of 2.5 μM CDDO or CDDO-Me led to the activation of caspase-9, which occurred after 6-hours treatment in RKO cells. As reported in Supplemetary Figure 3A, activated caspase-3/7 was detected by flow cytometry and showed a major increase after 16 and 24 hours of treatment, both for CDDO and CDDO-Me (Supplementary Figure S3B).

Concerning ROS, the gating strategy is reported in Figure 1F. Here, we reported a representative dot plot showing AnxV and TOPRO fluorescences to discriminate viable and dead cells, together with the histograms showing mitoPY1 and mitoSOX fluorescences in AnxV-/TOPRO- cells indicating the content of mtH_2_O_2_ and mtO_2_^−^ in living cells. In this figure, RKO cells were treated with H_2_O_2_, a molecule known to induce oxidative stress. The quantification of mitoPY1 and mitoSOX is reported in Figure 1G and 1H for RKO cells, and in Supplementary Figure S4A and S4B for HepG2 cells. In RKO cells, when CDDO was used, an increase in mtH_2_O_2_ and mtO_2_^−^ was in part observed at 6 hours, but especially after 16 hours of incubation (Figure 1G); when CDDO-Me was used, mtH_2_O_2_ and mtO_2_^−^ increased at 2.5 μM after 16 hours of incubation (Figure 1H). A slight increase after 24 hours of incubation was also observed. In HepG2 cells, CDDO led to a two to five-fold increase in mtH_2_O_2_ after 6, 16 and 24 hours of treatment. A parallel increase in mtO_2_^−^ levels was observed after 6 and 16 hours of incubation (Supplementary Figure S4A). When RKO cells were treated with CDDO-Me, the increase of mtH_2_O_2_ and mtO_2_^−^ was not observed after 6 hours of treatment but only after 16 hours in the presence of 2.5 μM CDDO-Me (Figure 1G). No change of mtH_2_O_2_ and mtO_2_^−^ was observed in HepG2 cells treated with CDDO-Me (Supplementary Figure S4B). We also investigated whether triterpenoids could increase mitochondrial ROS (mtROS) in non-transformed cells, *i.e*. primary fibroblasts, and we found that CDDO and CDDO-Me induced ROS generation but at lower levels compared to RKO and HepG2 cells (Supplementary Figure S4C and S4D). While in RKO and HepG2 cells after CDDO treatment, mtH_2_O_2_ had a 3- to 5-fold increase, in primary fibroblasts 10 μM CDDO was the only concentration able to induce a 1.5-fold increase of mtH_2_O_2_ (Supplementary Figure S4C, left panel). A similar, or even lower, increase was observed for mtO_2_^−^ (Supplementary Figure S4C, right panel).

### CDDO and CDDO-Me depolarize mitochondria and alter mitochondrial morphology

**Figure 2:**
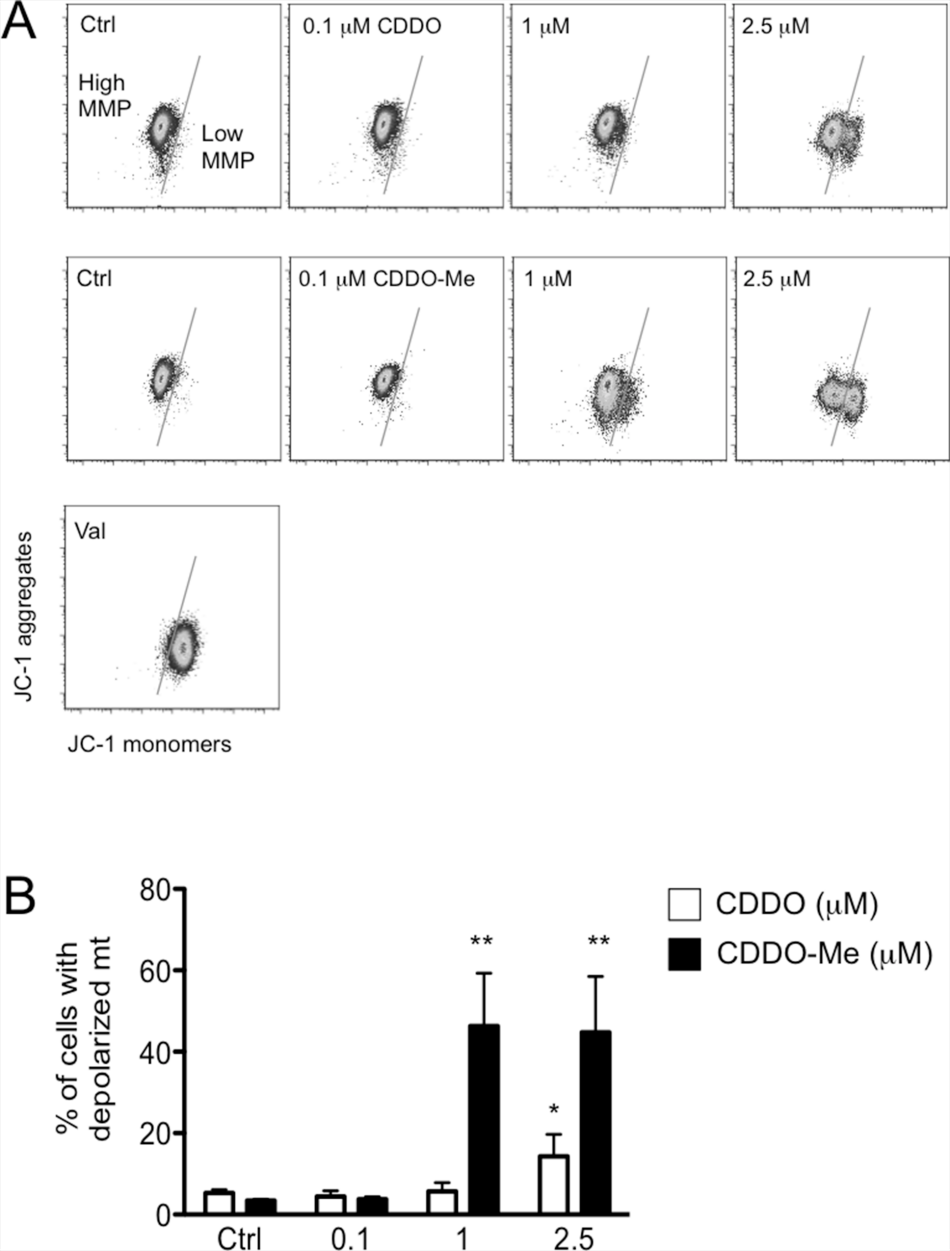
CDDO and CDDO-Me alter mitochondrial membrane potential (MMP) **A.** Representative dot plots showing JC-1 monomers and aggregates in cells treated with DMSO (Ctrl) and increasing concentrations of CDDO or CDDO-Me. Valinomycin (Val) was used a positive control for mitochondrial depolarization. **B.** Quantification of cells with depolarized mitochondria. Values represent the mean ± SD of five independent experiments, *P < 0.05 and **P < 0.01 *vs*. Ctrl.

We then quantified the amount of cells with depolarized mitochondria after 24 hours of culture in the presence of CDDO or CDDO-Me. In Figure 2A, representative dot plots showing JC-1 fluorescence are reported, whereas in Figure 2B and Supplementary Figure S5A the quantification of cells with depolarized mitochondria is reported for RKO and HepG2 cells, respectively. When CDDO was used, the concentration of 2.5 μM was sufficient to observe the presence of RKO cells with depolarized mitochondria, but higher concentrations were required to obtain a similar effect in HepG2 cells. Also in this case, CDDO-Me was much more potent, since the concentration of 1 μM was sufficient to cause a significant increase of cells with depolarized mitochondria both in RKO and in HepG2 cells. It is to note that concentrations higher than 2.5 μM caused massive cell death, so that the measure of mitochondrial membrane potential (MMP) had little meaning.

**Figure 3:**
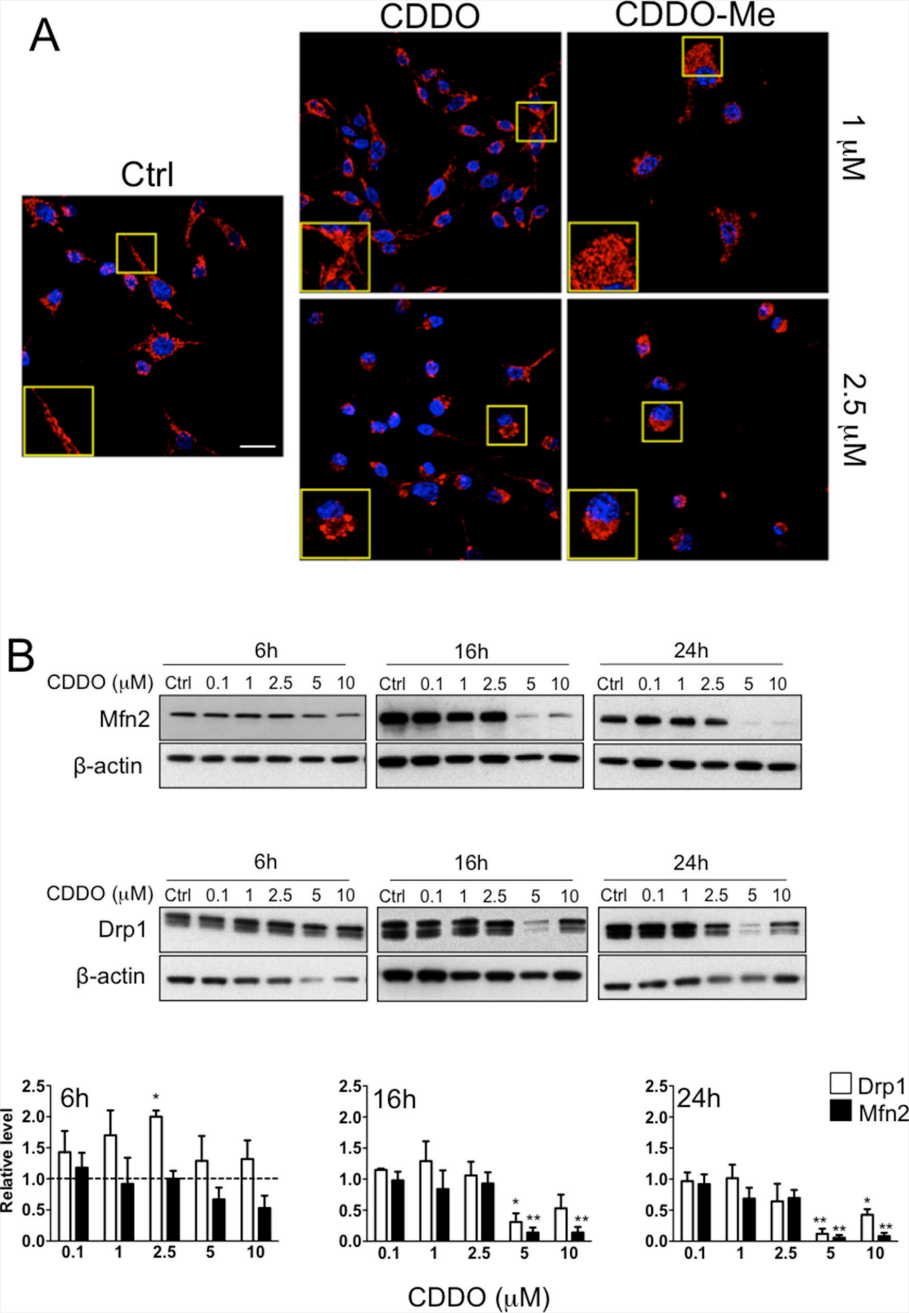

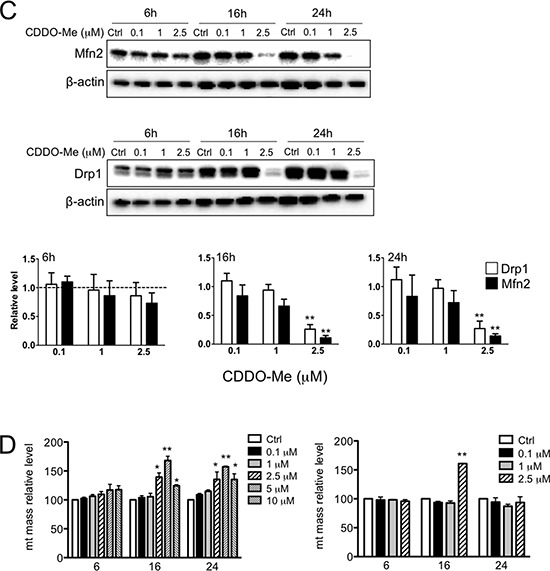
CDDO and CDDO-Me affect mitochondrial morphology and mitochondrial dynamics **A.** Representative immune-fluorescence images showing mitochondria in RKO cells treated with 1 μM and 2.5 μM CDDO and CDDO-Me. Red fluorescence represents mitochondria labelled with anti-human mitochondrial protein and with goat anti-rabbit F(ab’)_2_ Alexa 647. Nuclei were counterstained with DAPI. **B.** Representative western blot of mitofusin 2 (Mfn2) and dynamin related protein 1 (Drp1) in RKO cells treated with CDDO up to 10 μM for up to 24 hours. Densitometric data are also reported; values represent the mean ± SD of three independent experiments; *P < 0.05 and **P < 0.05 vs. Ctrl (dashed lines). **C.** Representative western blot of Mfn2 and Drp1 in RKO cells treated with CDDO-Me up to 2.5 μM for up to 24 hours. Densitometries are also reported; values represent the mean ± SD of three independent experiments; *P < 0.05 and **P < 0.01 *vs*. Ctrl (dashed line). **D.** Detection of mitochondrial mass in RKO treated with DMSO (Ctrl) and increasing concentrations of CDDO and CDDO-Me for up to 24 hours, as revealed by flow cytometry. Data are expressed as percentage of increase in median fluorescence intensity (MFI) in comparison to Ctrl, set to 100%, and represent the mean ± SD of four independent experiments; **P < 0.01 *vs*. Ctrl.

Changes in ROS production and MMP were accompanied by alteration of mitochondrial morphology and mitochondrial mass. Cells treated with CDDO and CDDO-Me contained fragmented mitochondria, that were present also at 1 μM concentration in all cell lines (Figure 3A and Supplementary Figure S5B). The expression levels of mitofusin-2 (Mfn2) and Dynamin related protein-1 (Drp1) were measured by western blot in lysates of RKO cells incubated with different concentrations of CDDO and CDDO-Me for 6, 16 and 24 hours. In RKO cells incubated with CDDO for 6 hours, Drp1 expression increased compared to untreated cells (Figure 3B). This increase is statistically significant at 2.5 μM. On the contrary, Mfn2 expression decreased compared to untreated cells especially at high drug concentrations (5 and 10 μM; Figure 3B). After 16 and 24 hours of treatment at high CDDO concentrations (5 and 10 μM), a significant decrease of both Drp1 and Mfn2 expression compared to untreated cells (Figure 3B) was found. A partial recovery of Drp1 was observed at 10 μM concentration (Figure 3B). As far as CDDO-Me treatment is concerned, after 16 and 24 hours of treatment, Drp1 and Mfn2 expression levels decreased in particular at high drug concentrations (1 and 2.5 μM; Figure 3C).

Both drugs altered mitochondrial mass, in particular after 16 and 24 hours of treatment with CDDO both in RKO and in HepG2 cells (Figure 3D and Supplementary Figure S5C). Slight modifications in mitochondrial mass were observed with CDDO-Me. Similar alterations in MMP and mitochondrial morphology were observed in MCF7 cells (Supplementary Figure S6A and S6B).

### CDDO and CDDO-Me alter the expression of Lon targets, and Lon overexpression protect cells from apoptosis induced by these molecules

**Figure 4:**
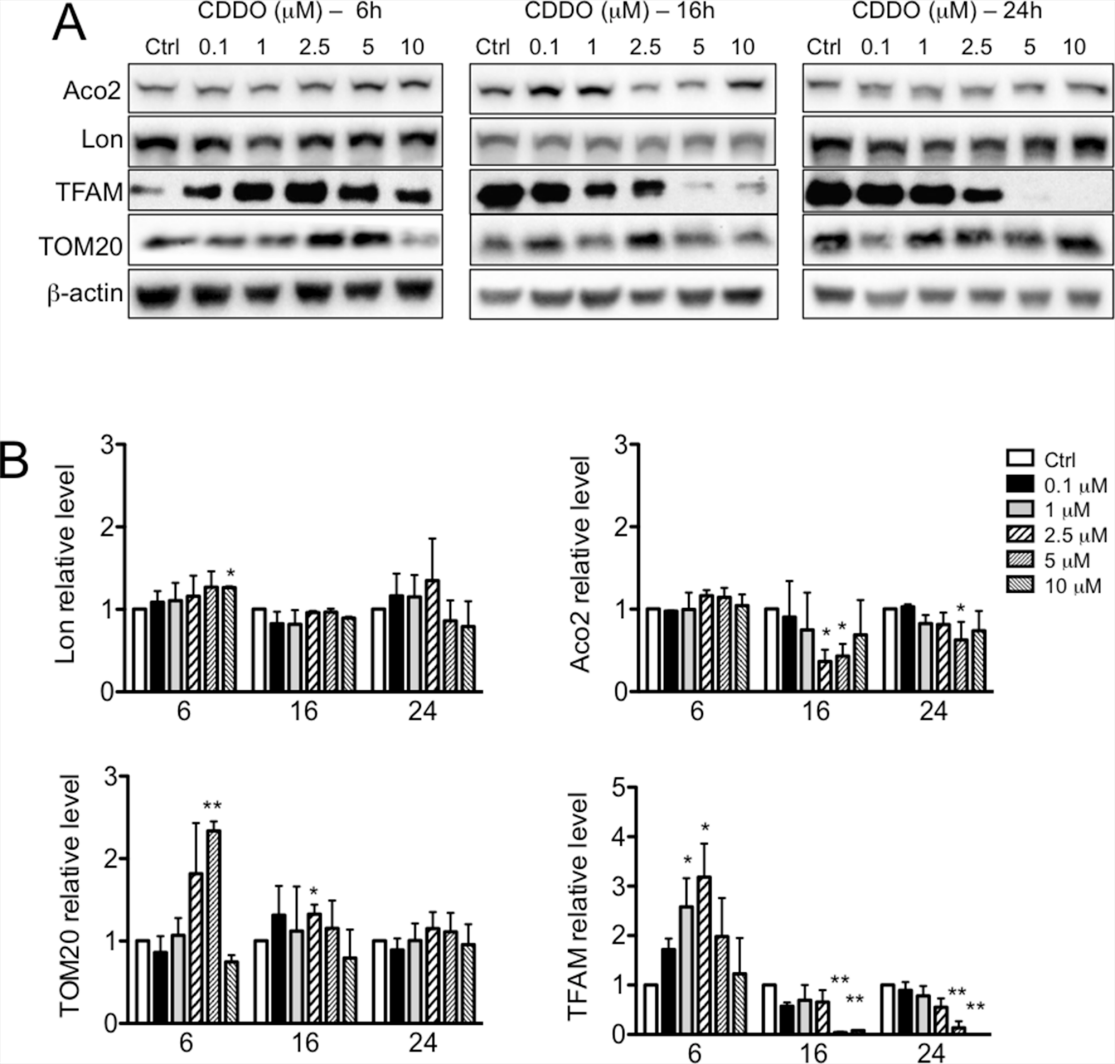

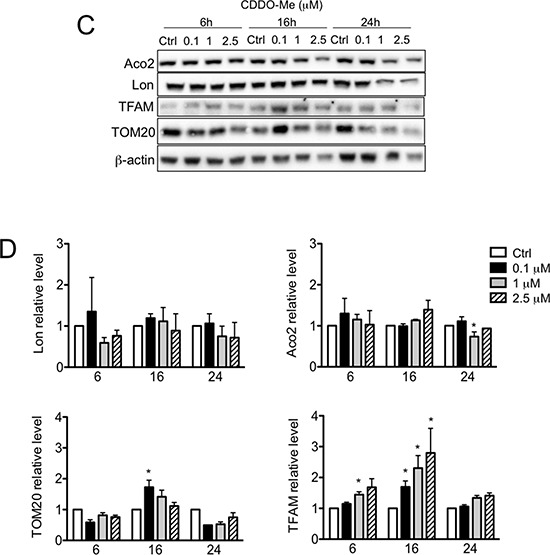
Effects of CDDO and CDDO-Me on the expression of Lon protease and its proteolytic targets **A.** Representative western blots of Lon, human mitochondrial transcription factor A (TFAM) and aconitase (Aco2), and TOM20 in RKO cells treated with increasing concentrations of CDDO for up to 24 hours. β-actin were used as loading control. **B.** Densitometric analysis showing the levels of Lon, TFAM, Aco2 and TOM20 in CDDO-treated cells. Values represent the mean ± SD of three independent experiments, *P < 0.05 *vs*. Ctrl. **C.** Representative western blots of Lon, TFAM, Aco2 and TOM20 in RKO cells treated with increasing concentrations of CDDO-Me for 6, 16 and 24 hours. **D.** Densitometric analysis showing the levels of Lon, TFAM, Aco2 and TOM20 in cells treated with CDDO-Me. Values represent the mean ± SD of three independent experiments, *P < 0.05 *vs*. Ctrl.

We next examined whether and to what extent the level of Lon and of two representative substrates, namely human transcription factor A (TFAM) and aconitase (Aco2) were modified after treatment with CDDO or CDDO-Me. Lon protein levels remained almost unchanged (Figure 4A and 4B, left panel); a slight reduction of Lon mRNA was observed after 16 hours of treatment, which was maintained until 24 hours (Supplementary Figure S7). Concerning CDDO-Me, Lon mRNA showed only a slight reduction after 16 hours of treatment with 2.5 μM CDDO-Me. Aco2 protein levels slightly decreased after 16 hours incubation with CDDO, whereas TFAM protein levels increased after 6 hours of treatment and then strongly decreased at the higher concentrations of CDDO after 16 and 24 hours of treatment (Figure 4A and 4B). Similarly, when cells were treated with CDDO-Me, Aco2 levels did not change, whereas TFAM levels increased after 16 hours of treatment (Figure 4C and 4D).

**Figure 5:**
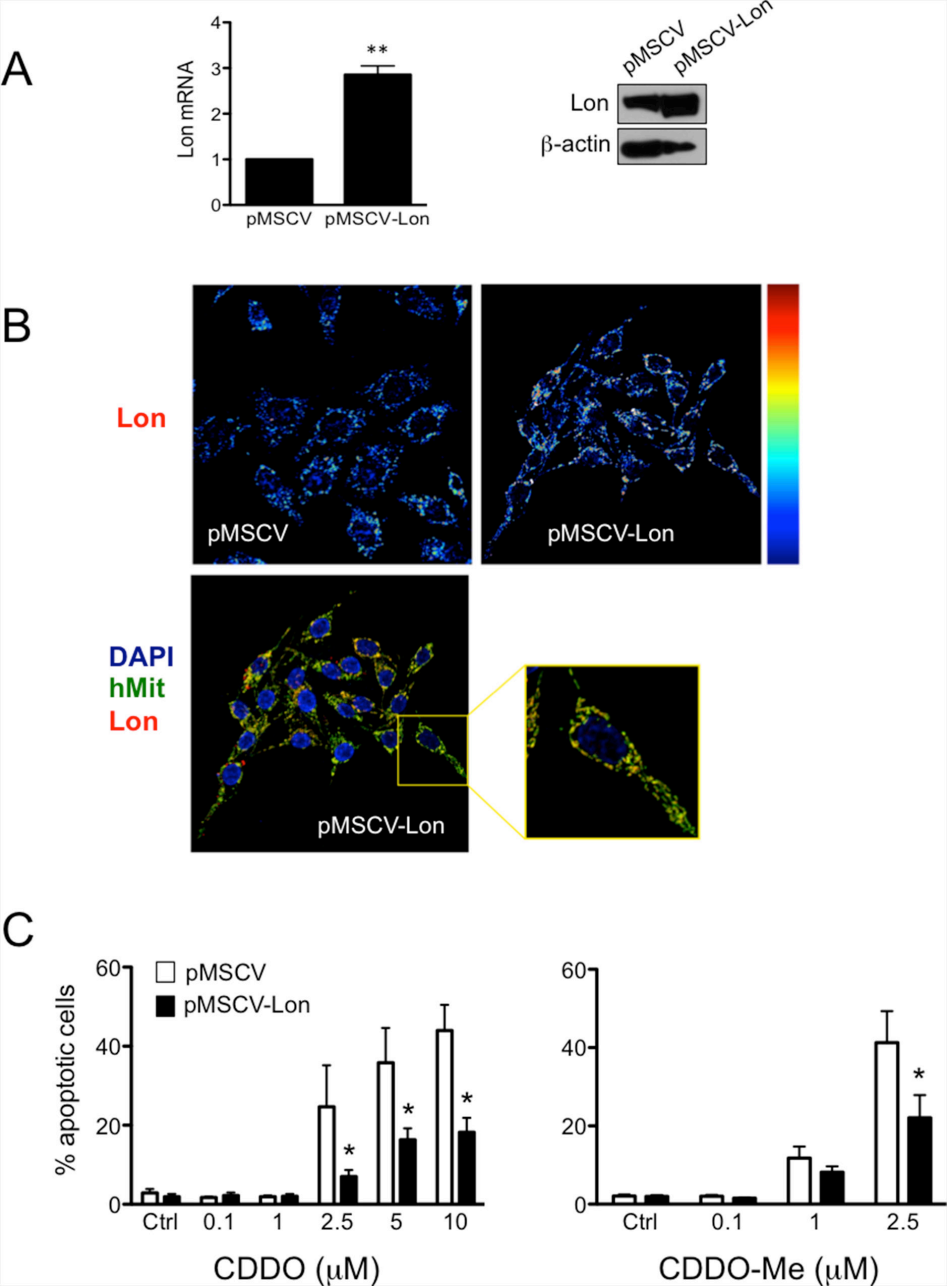
Overexpression of Lon protects cells from apoptotic cell death induced by CDDO and CDDO-Me **A.** Expression of Lon mRNA and Lon protein in control cells (pMSCV) and cells overexpressing Lon (pMSCV-Lon). Values represent the mean ± SD of five independent experiments, **P < 0.01 *vs*. Ctrl. **B.** Representative pseudocolour maps (upper panels; red indicates maximal expression) obtained by confocal microscopy after immunostaining cells with anti-Lon antibody. In lower panels, representative confocal microscopy images show that Lon is localized to mitochondria in pMSCV-Lon cells. Cells were immunostained with anti-Lon (red) and anti-human mitochondria (hMit, green). Nuclei were counterstained with DAPI. **C.** Percentage of apoptotic cells after treatment with CDDO up to 10 μM, and CDDO-Me up to 2.5 μM. Values represent the mean ± SD of five independent experiments, *P < 0.05 *vs*. Ctrl.

Considering that Lon is a stress protein inducible by multiple stressors, we asked whether Lon overexpression could protect RKO cells from CDDO-induced cell death. Thus, we generated a retroviral vector harbouring the cDNA encoding for Lon protease (pMSCV-Lon), and we used this to stably transduce RKO cells. We firstly ascertained that cells transduced with pMSCV-Lon had increased levels of Lon mRNA and Lon protein. In pMSCV-Lon cells, a 3-fold increase and a 2-fold increase was observed for Lon mRNA and Lon protein, respectively (Figure 5A). We also confirmed that Lon was correctly localized to mitochondria in these cells (Figure 5B). When treated with CDDO and CDDO-Me, cells overexpressing Lon were less prone to undergo apoptosis than control cells (Figure 5C). Interestingly, also in this case CDDO-Me was more effective than CDDO in causing cell death.

### CDDO and CDDO-Me increase the levels of mitochondrial protein carbonyls

**Figure 6:**
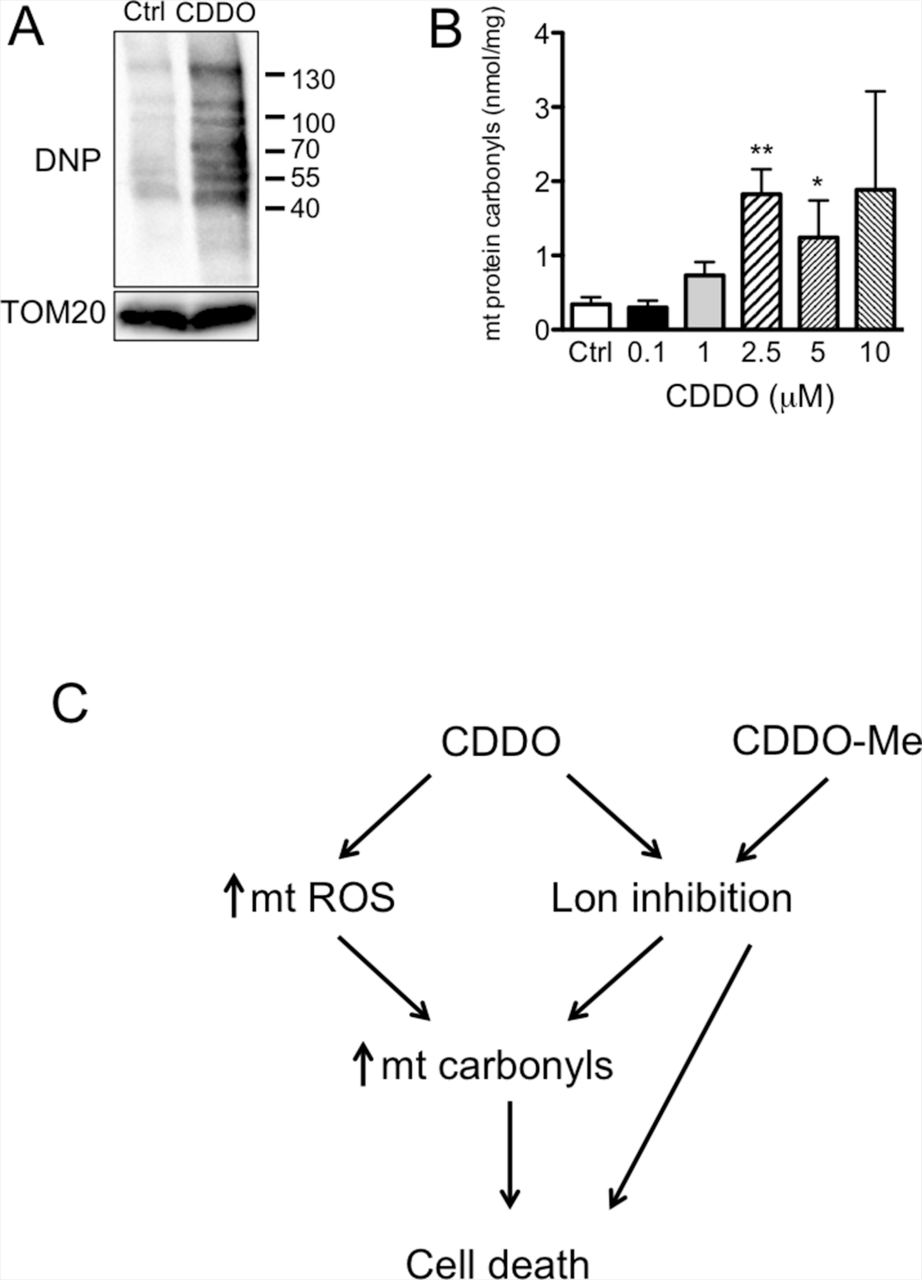
CDDO and CDDO-Me increased mitochondrial protein carbonyls **A.** Detection of protein carbonyls in mitochondrial lysates, as revealed by oxyblotting by using a primary antibody against dinitrophenyl hydrazone (DNP). RKO cells were treated with 2.5 μM CDDO for 16 hours. **B.** Quantification of protein carbonyls in mitochondrial fractions from RKO cells treated with CDDO at different concentrations for 16 hours, as revelaed by colorimetric assay. Values represent the mean ± SD of three independent experiments, *P < 0.05 and **P < 0.01 *vs*. Ctrl. **C.** Schematic representation of the possible mechanisms linking CDDO and CDDO-Me to cell death.

As protein carbonylation is the main protein modification that takes place as consequence of severe oxidative stress, we investigated whether the increase in mtROS observed in RKO cells treated with CDDO and CDDO-Me determined an increase in the levels of protein carbonyls in mitochondria. Protein carbonylation was assessed in mitochondrial fractions by derivatization of carbonyls with 2, 4-dinitrophenylhydrazine (DNPH), followed by antibody detection. In Figure 6A a representative oxyblot performed on mitochondrial fractions from RKO cells treated with 2.5 μM CDDO is reported. As expected, the levels of protein carbonyls, as revealed by colorimetric assay, were significantly increased (Figure 6B). CDDO-Me led to a slight, but non significant increase at the highest tested concentration (data not shown).

## DISCUSSION

Our previous studies demonstrated that mitochondrial Lon protease plays a crucial role in maintaining mitochondrial morphology and function in RKO colon carcinoma cells [13]. We showed that Lon is up-regulated in several cancer cell lines and in tissue samples from colorectal carcinoma, and that in RKO cells Lon down-regulation leads to impaired mitochondrial proteome, reduced levels of mitochondrial RNAs, reduced oxygen consumption rate, increased ROS and fragmented shape of mitochondrial network. In these cells, apoptotic cell death occurred by the intrinsic pathway, with release of cytochrome *c* in the cytosol, and activation of caspase-9 and PARP. These features potentially place Lon among non-oncogenic proteins, that is those proteins which do not initiate tumourigenesis, but are important for cancer cell survival. For this reason, the current study was aimed at determining the effects of two inhibitors of Lon proteolytic activity, the triterpenoids CDDO and CDDO-Me, in different cancer cells. In recent years, the pharmacologic action of CDDO and CDDO-Me gained increased attention, and several studies reported their antiproliferative and anticancer activities. We confirmed that CDDO and CDDO-Me inhibited cell growth in a concentration-dependent manner and induced apoptosis in RKO, HepG2 and MCF7 cells. In particular, the fact that apoptosis mediated by CDDO and CDDO-Me was identified by an increase in phosphatidylserine exposure, and by the activation of caspase-3, together with the activation of caspase-9, places these triterpenoids among molecules able to exert toxic effects on mitochondria.

As previous studies have shown that triterpenoids induce apoptosis as a consequence of ROS generation [20, 21], we analysed mitochondrial redox state and MMP. We found that CDDO leads to increased mtH_2_O_2_ and mtO_2_^−^ production, particularly after 16 hours of treatment, and determines an increase of carbonylated mitochondrial proteins. The capability of this agent to increase oxidative stress could be direct, for instance through the interaction with electron transport chain complexes, or indirect, through the inhibition of Lon, which protects mitochondria from stresses and from the accumulation of oxidized and carbonylated proteins. Complexes I and III of the mitochondrial electron transport chain are major sites where superoxide is generated, either in the matrix (Complex I and III), or in the intermembrane space (Complex III) [22]. Superoxide can be partially converted into hydrogen peroxide, which can be scavenged by antioxidant systems, including catalase, peroxiredoxins and glutathione [23]. When ROS accumulate, they can oxidize proteins in a process known as protein carbonylation, which is the irreversible, metal-catalyzed oxidative attack on the side chains of precise aminoacids [24]. If not degraded, carbonylated proteins form high-molecular-weight cytotoxic aggregates. As Lon is responsible for degradation of carbonylated proteins in mitochondrial matrix, the increase of carbonylated proteins could be likely due to the sum of two concomitant effects of CDDO and CDDO-Me: the capability to increase the levels of mtROS, which in turn determines mitochondrial protein carbonylation, and the inhibition of Lon, which is responsible for the degradation of carbonylated proteins. Thus, the pro-apoptotic and cytotoxic activity of these two triterpenoids can be due to the direct effect of ROS, which are pro-apoptotic *per se*, to damage induced by ROS on mt proteins, and to the inhibition of Lon that lowers the removal of cytotoxic protein aggregates within mitochondria.

Cancer cells produce higher levels of ROS than normal cells [25, 26]. On the one hand, these ROS play an important role in cancer progression by stimulating cell growth and genetic instability. On the other, they generate an intrinsic oxidative stress which renders cells highly dependent on antioxidant systems and/or highly sensitive to agents that dysregulate redox status. For instance, the association of 2-methoxyestradiol (2-ME), which inhibits superoxide dismutase, to arsenic trioxide, which causes ROS increase, has a strong cytotoxic activity in leukemia cells [27]. Similarly, hydrogen peroxide and 2-ME can induce oxidative stress that leads to apoptotic and autophagic cell death in transformed cells but not in normal, non-transformed cells [28]; finally, β-phenylethyl isothiocyanate selectively kills cancer cells through a ROS-mediated mechanism [29]. Our data indicate that CDDO and CDDO-Me exert a similar effect, as they increase mitochondrial oxidative stress, which contributes to apoptotic cell death in cancer cells, but fail to induce mtROS increase and apoptosis in normal fibroblasts. We observed that CDDO and CDDO-Me severely altered mitochondria of RKO, HepG2 and MCF7 cells, without deeply affecting the levels of Lon protein and mRNA, the levels of the main substrates of Lon proteolytic activity (except for TFAM), and the levels of proteins involved in mitochondria fusion and fission, except for the highest concentrations of CDDO and CDDO-Me.

Mitochondrial morphology is regulated by the balance of two continuous antagonistic processes: fusion and fission [30]. Under physiological conditions, mitochondria are elongated and filamentous, but they undergo extensive fragmentation during apoptosis. It is generally believed that increased mitochondrial fragmentation signals cellular damage and initiates apoptotic death of the cell and that an unbroken fusion process, on the other hand, may lead to uncontrolled proliferation [31]. As vital determinants of fission-fusion balance, Drp1 and Mfn2 share a reciprocal relationship. Factors perturbing this balance are likely to also skew the stoichiometric relationship between these two proteins [32]. Apoptotic fission is associated with remodeling of the cristae, which is characterized by the opening of their tubular junction and release of proapoptotic factors, such as cyt *c*, which is required in the cytosol for the activation of downstream effector caspases [33]. In RKO cells, after 6 hours of treatment the Drp1/Mfn2 ratio increased and this unbalance might promote mitochondrial fragmentation and stimulate apoptotic death that is highlighted after 16 and 24 hours of treatment. After 16 hours of treatment Drp1 and Mfn2 expression levels decreased in particular at high drug concentrations (5 and 10 μM). Nevertheless, Drp1/Mfn2 ratio remained unbalanced towards fission. At 24 hours of treatment Drp1 and Mfn2 expression decreased in particular at high concentrations of CDDO. The reduced expression level of these proteins might be the consequence of no more fully sustained *de novo* protein synthesis at these high drug concentrations and can explain the disruption of mitochondria in terms of morphology. In fact, Mfn2 is essential for maintaining mitochondrial shape and integrity and alteration of protein synthesis of Drp1 may alter mitochondrial turnover (mitophagy) [30]. A partial recovery of Drp1 was observed at 10 μM concentration after 16 and 24 hours of incubation. This result may be explained as the last attempt of the cells to increase mitochondrial functionality by activating mitochondrial fission and mitochondrial turnover to remove mitochondrial cytotoxic protein aggregates. High CDDO concentration (10 μM) may inhibit Lon protease and increase protein aggregates. In the presence of CDDO-Me, again, the reduced expression of Drp1 and Mfn2 might be the consequence of no more fully sustained *de novo* protein synthesis and can explain the disruption of mitochondrial morphology. No increase of Drp1 expression was observed in CDDO-Me treated cells. It can be envisioned that mitochondrial fragmentation and apoptosis in CDDO-Me treated cells might be activated by a different mechanism such as the increase of another fission protein like Fis1 [34] or the depletion of another fusion protein like OPA1 [35].

Strong differences were observed in the effects of CDDO and CDDO-Me on oxidative stress and mitochondrial mass. In our models, CDDO induced a burst of ROS specifically in mitochondria, which resulted in mitochondrial damage, collapse of MMP, mitochondrial mass increase and cell death by intrinsic apoptosis. Conversely, CDDO-Me was not associated with mtROS increase (at least not in a concentration- and time-dependent mechanisms as was for CDDO); in any case, it induced mitochondrial damage, collapse of MMP and apoptotic cell death without altering mitochondrial mass. Interestingly, several reports show that mitochondrial mass increases in the presence of oxidative stress and that such increase depends on MMP and protein synthesis in the cytoplasm [36, 37]. A similar increase has also been observed in different cell types undergoing apoptotic cell death, including HepG2, MCF7 and HeLa cells [38, 39]. The reasons at the basis of the increase in mitochondrial mass are scarcely known, but it is plausible that it could represent a response to stress in order to enhance energy supply.

We have found that Lon inhibition by CDDO and CDDO-Me alters mitochondrial morphology and functionality, and in turn this impairment determines a higher production of ROS in mitochondria, and oxidative stress. The protein levels of Lon were not strongly influenced by CDDO or CDDO-Me treatment whereas the levels of TFAM and Aco2 were slightly modulated; this indicates that high concentrations are likely able to block Lon functions even when this protein is synthetized at higher levels, and cells could not avoid loss of mitochondrial functionality, accumulation of carbonylated, cytotoxic proteins, and cell death.

In conclusion, we have demonstrated that CDDO and CDDO-Me act through a pathway that involves mitochondria and mitochondria-specific oxidative stress; such triterpenoids induce apoptosis in cancer cells, but not in normal, non-transformed cells. The association between their capability to increase mtROS production and to inhibit Lon proteolytic activity probably renders these agents good candidates for anticancer therapy (Figure 6C). Finally, the fact that the overexpression of Lon reduces apoptotic cell death induced by CDDO and CDDO-Me indicates that Lon is a key non-oncogenic molecule for the maintenance of mitochondria functions in cancer cells.

## MATERIALS AND METHODS

### Cell culture and reagents

RKO cells were cultured in RPMI Glutamax supplemented with 10% foetal bovine serum (FBS) and gentamycin. HepG2 cells were cultured in MEM supplemented with 10% FBS, 1% non essentials aminoacids, 1% vitamins, 1% L-glutamine, and gentamycin. MCF7 cells were cultured in Dulbecco's Modified Eagle Medium (DMEM) Glutamax supplemented with 10% FBS and gentamycin. Human fibroblasts were obtained after informed and signed consent from 1 healthy subject (40 years old) and used between 5^th^ and 7^th^ passages. Fibroblasts were isolated and established in culture according to standard methods [40]. Fibroblasts were grown in 75 cm^2^ flasks for 5 days in DMEM supplemented with 10% FBS, until confluence. Then, 1.2 × 10^5^ cells were seeded in 6-well plate and treated with CDDO or CDDO-Me when confluent. Cells were maintained in 5% CO_2_ atmosphere at 37°C. CDDO and CDDO-Me were purchased from Cayman Chemical Company (Ann Arbor, Michigan, USA), were dissolved in DMSO and were used at concentrations up to 10 μM. All culture media and culture reagents were from Life Technologies Corporation (Carlsbad, CA, USA).

### Retroviral transduction

The pMSCV-Puro empty vector and the pMSCV containing the cDNA encoding for Lon protease (pMSCV-Lon) were used to transiently transfect amphotrophic Phoenix (phxA) cells, and the retroviral transduction has been performed as follows. PhxA cells were seeded and transfected. Transient transfection was performed by using lipofectamine 2000 (Life Technologies Corporation) and 20 μg of pMSCV-Lon plus 0.9 μg of helper plasmid. PhxA cells were maintained in the presence of transfection mix for 16 hours, then switched to 20 mL of RKO medium and placed in the incubator for 24 hours. At the end of the incubation, the retroviral supernatant was collected, filtered with 0.45 μm filter and supplemented with 8 μg/mL hexadimethrine bromide, and used to stably transfect RKO cells. RKO cells were seeded in 6-well plates at density of 0.5 × 10^5^ cells/mL. Two mL of retroviral supernatants was added to RKO cells that were then centrifuged at 1,800 rpm at 32°C for 45 minutes. Cells were then placed in the 32°C incubator. After 2 hours, medium was replaced with fresh retroviral supernatants and centrifuged as indicated before. Cells were then placed in the 32°C incubator for 4 hours. At the end of incubation, normal medium was added to substitute retroviral supernatant, and cells incubated overnight. The day after, normal medium was removed and replaced with 4 mL of fresh retroviral supernatant, and cells were centrifuged at 1,800 rpm at 32°C for 45 minutes. Cells were then put at 32°C for 5 hours, and retroviral supernatants was replaced with normal medium. After 48 hours, 2 μg/mL puromycin was added to the cells. RKO cells which survived the selection were maintained in medium supplemented with 1 μg/mL puromycin.

### Flow cytometric analysis

The analysis of apoptosis, mitochondrial H_2_O_2_ and mitochondrial O_2_^−^ was performed by staining cells with Annexin-V Alexa Fluor Pacific Blue conjugate (AnxV, Life Technologies Corporation), TO-PRO-3 iodide (TOPRO, Life Technologies Corporation), mitochondria Peroxy Yellow 1 (mitoPY1, Sigma Aldrich, St. Louis, MO, USA), and MitoSOX Red Superoxide Indicator (MitoSOX, Life Technologies Corporation). Cells were first loaded with 10 μM mitoPY1 in complete medium for 60 minutes and then treated with CDDO or CDDO-ME for up to 24 hours [41]. As a positive control, cells were treated with 1mM H_2_O_2_ for 1 hour. At the end of the incubation, cells were washed with PBS and incubated with 5 μM MitoSOX in complete medium, for 30 minutes at 37°C. Then, cells were stained with AnxV in Annexin-V binding buffer (10 mM HEPES, 140 mM NaCl, 2.5 mM CaCl_2_, pH 7.4) for 10 minutes in the dark. TOPRO was added before acquisition to assess membrane integrity of cells. Samples were analyzed by flow cytometry. In particular, cells were first gated on the basis of physical parameters. Then, in living cells (AnxV-/TOPRO- cells) the median fluorescence intensity (MFI) of mitoPY1 and MitoSOX was analysed. The MFI of the unstained sample was subtracted to the MFI of the stained sample to avoid any influence related to the eventual autofluorescence. Then, the net MFI value obtained for control sample was set to 100 and other samples calculated according the following formula: Relative MFI level = (treated sample net MFI/untreated sample net MFI)x100.

Mitochondrial membrane potential was analysed by staining cells with 5,5′,6,6′-tetra-chloro-1,1′,3, 3′-tetraethylbenzimidazolyl-carbocyanine iodide (JC-1, Life Technologies Corporation) [42]. Briefly, cells were stained with 2.5 μM JC-1 in complete medium, and incubated for 10 minutes in the dark; valinomycin was used as positive control [43]. Mitochondrial mass was evaluated by staining cells with MitoTracker Green (Life Technologies Corporation) [44]. Cells were incubated with 200 nM MitoTracker Green in culture media for 30 minutes at 37°C, and then analysed.

The activation of caspase-3 was evaluated by using the CellEvent Caspase-3/7 Green Detection Reagent (CellEvent, Life Technologies Corporation), which consists of a four amino acid peptide, which is intrinsically non-fluorescent and emits fluorescence when cleaved by caspase-3/7 in apoptotic cells. Briefly, cells were treated with CDDO or CDDO-Me. Then, supernatants were collected, cells were trypsinized, and stained with 5 μM CellEvent for 30 minutes, and then analyzed.

All samples were analysed by using an Attune Nxt Acoustic Focusing Cytometer (Life Technologies Corporation). The instrument is equipped with a blue laser (488 nm), a red laser (637 nm), a yellow laser (561 nm) and a violet laser (405 nm) [45]. Data were acquired in list mode using Attune Cytometric 2.1 software, and then analysed by FlowJo 9.8.5 (Tree Star Inc., Ashland, OR, USA) under Mac OSX.

### Western blot analysis

Total cellular proteins were obtained by lysing cells with RIPA buffer (50 mM Tris, pH 7.5, 0.1% Nonidet P-40, 0.1% deoxycholate, 150 mM NaCl, 4 mM EDTA, and protease inhibitors cocktail). Then, 10 to 50 μg of total cellular protein or mitochondrial fraction, were subjected to SDS-PAGE in 4–12% or 12% precast gels (Life Technologies Corporation) followed by transfer to the proteins onto a nitrocellulose membrane (Bio-Rad Laboratories, Hercules, CA, USA) and immunoblotting. The following antibodies were used: anti-cleaved Caspase-9 (Cell Signaling Technology, Danvers, MA, USA), anti-Lon (Primm, Milan, Italy), anti-TFAM (Abnova Corporation, Taipei, Taiwan), anti-Aco2 (Cell Signaling Technology), anti-TOM20 (Santa Cruz Biotechnology, Dallas, Texas, USA), anti-MFN2 (Abnova Corporation, Taipei City, Taiwan), anti-DNM1L (Abnova Corporation), goat anti-rabbit IgG (Fc):HRP (Serotec, Kidlington, UK). The blot was developed and images were captured by using a Chemidoc MP (Bio-Rad Laboratories). The densitometric analysis was performed using ImageLab 5.2.1 (Bio-Rad Laboratories). Oxyblot was performed by using the OxyBlot Protein Oxidation Detection Kit (Millipore, Billerica, MA, USA), following the manufacturer's instructions. Briefly, cells were collected, washed with PBS and resuspended in a cytosolic lysis buffer (250 mM sucrose, 70 mM KCl, 137 mM NaCl, 4.3 mM Na_2_HPO_4_, 1.4 mM KH_2_PO_4_ pH 7.2 and protease inhibitors) for 5 minutes on ice. Then cells were centrifuged at 1,000 g for 10 minutes at 4°C, and the pellet was resuspended in mitochondrial lysis buffer (50 mM Tris-HCl pH 7.4, 150 mM NaCl, 2 mM EDTA, 2 mM EGTA, 0.2% Triton X-100, 0.03% NP40 and protease inhibitors) for 5 minutes on ice. The suspension was centrifuged at 10,000 g for 10 minutes at 4°C and the supernatant was collected as the mitochondrial fraction. Two aliquots (15 μg) of each mitochondrial lysate were used: one was subjected to the derivatization reaction and the other was incubated with derivatization-control solution. Then, samples were loaded in a 4–15% precast gel (Bio-Rad Laboratories, Hercules, CA, USA), and an anti-DNP antibody was used for the detection of the DNPH-derivatized proteins.

### Immunofluorescence analysis of mitochondrial morphology

Cells were seeded at density of 2 × 10^5^ cells/mL on glass coverslips in 6-well plates. After treatment, cells were fixed with 4% paraformaldehyde for 10 minutes and permeabilised with 0.1% Triton X-100 in phosphate buffered saline (PBS) for 5 minutes, and blocked with 3% bovine serum albumin (BSA) in PBS for 30 minutes, at room temperature. Samples were incubated with anti-human mitochondrial protein (Millipore) in PBS containing 3% BSA, for 1 hour at room temperature. After washing in PBS containing 3% BSA, samples were incubated for 1 hour at RT with a goat anti-rabbit F(ab’)_2_ Alexa647 (Life Technologies Corporation). After washing in PBS, samples were stained with 1 μg/ml 4′,6-diamidino-2-phenylindole (DAPI) in PBS for 1 minute, and then mounted with anti-fading medium (0.21 M 1,4-Diazabicyclo [2.2.2] octane (DABCO) and 90% glycerol in 0.02 M Tris, pH 8.0). Negative controls were samples not incubated with the primary antibody. Then, samples were observed by a Nikon A1 confocal laser scanning microscope equipped with 4 lasers: a 405 nm diode laser for DAPI, a 488 nm argon laser, a 543 nm HeNe laser and a 637 nm diode laser. The excitation and the detection of the samples were carried out in sequential mode to avoid overlapping of the two signals. Optical sections were obtained at increments of 0.5 μm in the z-axis and were digitized with a scanning mode format of 1024 × 1024 pixels and 4096 gray levels. Spectral analysis was carried out to exclude overlapping between multiple signals. The confocal serial sections were processed with ImageJ software to obtain three-dimensional projections and image rendering was performed by Adobe Photoshop Software.

### Measurement of mitochondrial protein carbonyls

Protein carbonyls were measured by using the Protein Carbonyl Colorimetric Assay Kit (Cayman Chemical Company, Ann Arbor, MI, USA) according to manufacturer's instructions. Briefly, 400 μg of mitochondrial fraction were used to assay protein carbonyls. The carbonyl content was calculated by measuring the absorbance at 385 nm was normalized on the basis of protein concentration at 280 nm.

### Statistical analysis

The results are expressed as the mean ± SD. Significance was determined by paired *t* test, and *p* < 0.05 was considered statistically significant.

## SUPPLEMENTARY FIGURES


