# Supplementary material for: Inhibition of Lon protease by triterpenoids alters mitochondria and is associated to cell death in human cancer cells
Source: Oncotarget. 2015 Jul 15;6(28):25466–83. doi: 10.18632/oncotarget.4510 (PMC4694845; doi:10.18632/oncotarget.4510)
Supplement: Supplementary file 1 [file oncotarget-06-25466-s001.pdf]

SUPPLEMENTARY FIGURES

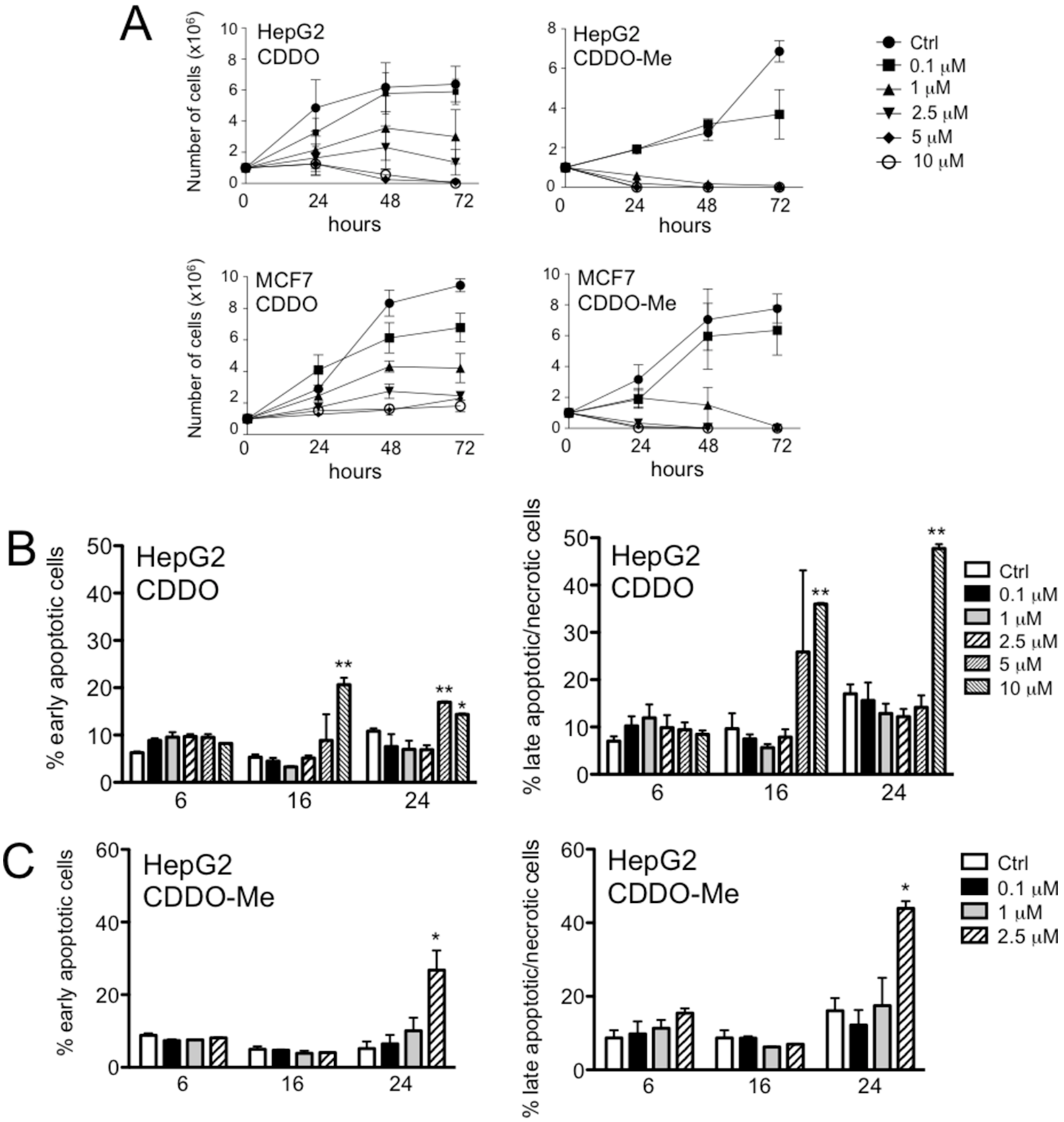

Supplementary Figure S1: CDDO and CDDO-Me are anti-proliferative and induce apoptosis in HepG2 and MCF7 cells. A. Growth curve of HepG2 and MCF7 cells incubated with the indicated concentrations of CDDO (left panel) and CDDO-Me (right panel) for up to 72 hours. Cells incubated with DMSO were used as controls (Ctrl). **B.** Percentage of early apoptotic cells and late apoptotic/necrotic cells after treatment with the indicated concentrations of CDDO for up to 24 hours. Values represent the mean ± SD of three independent experiments, *$P < 0.05$ and **$P < 0.01$ *vs*. Ctrl. **C.** Percentage of early apoptotic cells and late apoptotic/necrotic cells after treatment with the indicated concentrations of CDDO-Me for up to 24 hours. Values represent the mean ± SD of three independent experiments, *$P < 0.05$ and **$P < 0.01$ *vs*. Ctrl. (*Continued*)

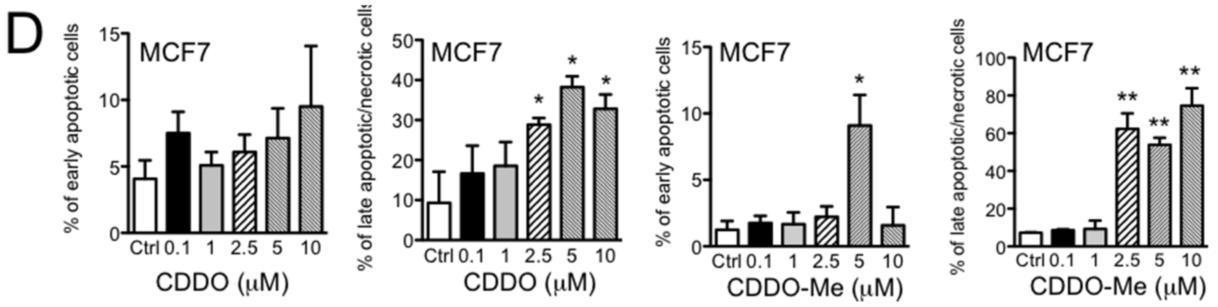

Supplementary Figure S1: (*Continued*) CDDO and CDDO-Me are anti-proliferative and induce apoptosis in HepG2 and MCF7 cells. D. Percentage of early apoptotic cells and late apoptotic/necrotic cells after treatment with the indicated concentrations of CDDO or CDDO-Me for 24 hours. Values represent the mean ± SD of three independent experiments, *$P < 0.05$ and **$P < 0.01$ *vs*. Ctrl.

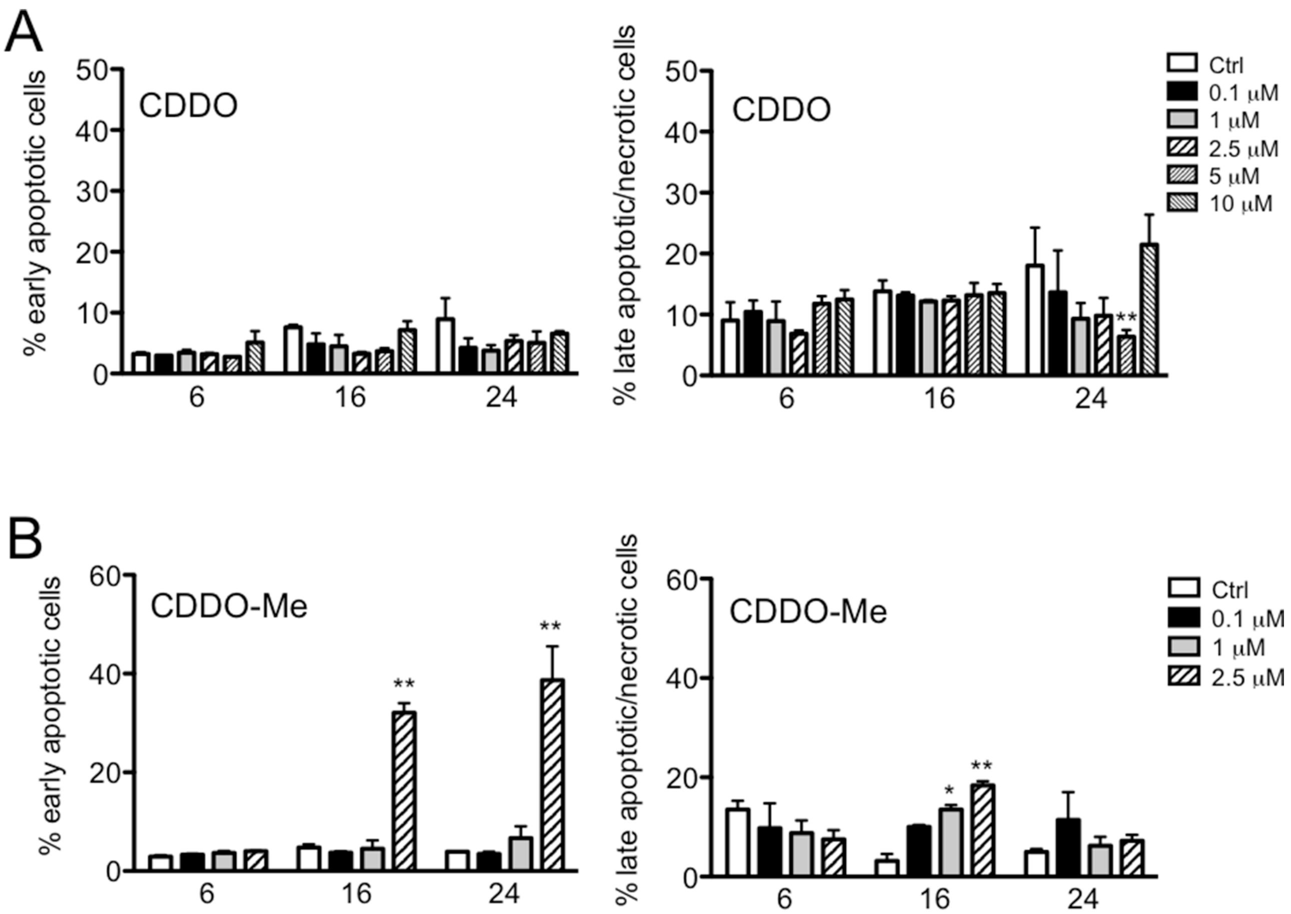

Supplementary Figure S2: CDDO and CDDO-Me do not induce apoptosis in primary human fibroblasts. A. Percentage of early apoptotic cells and late apoptotic/necrotic cells after treatment with CDDO for up to 24 hours. Values represent the mean ± SD of three independent experiments. **$P < 0.01$ *vs*. Ctrl **B.** Percentage of early apoptotic cells and late apoptotic/necrotic cells after treatment with CDDO-Me for up to 24 hours. Values represent the mean ± SD of three independent experiments, *$P < 0.05$ and **$P < 0.01$ *vs*. Ctrl.

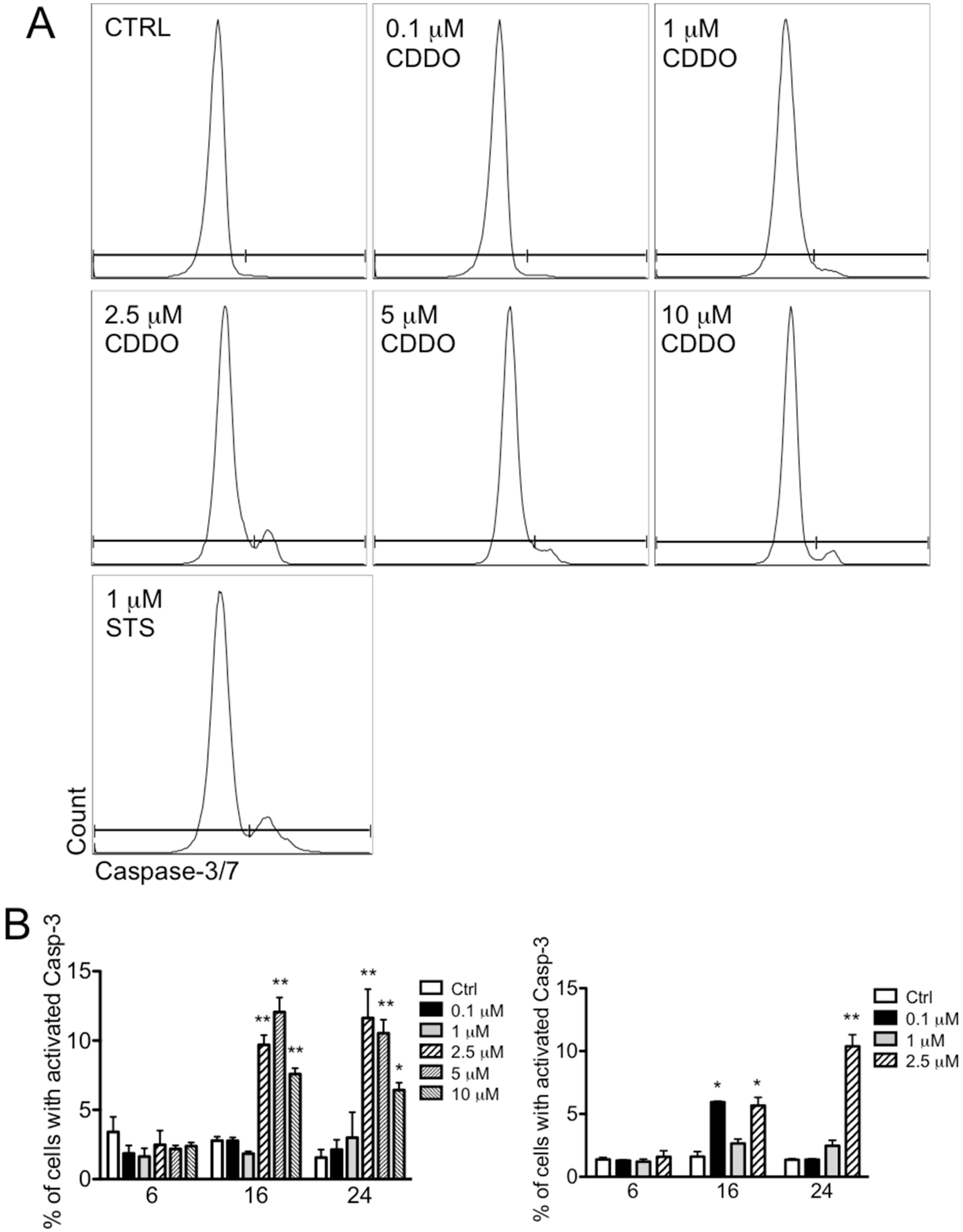

Supplementary Figure S3: CDDO and CDDO-Me lead to activation of caspase-3/7. A. Representative histograms showing CellEvent Caspase-3/7 Green Detection Reagent in RKO cells treated with increasing concentrations of CDDO, as revealed by flow cytometry. Cells treated with 1 µM staurosporine (STS) for 4 hours were used as positive control for caspase activation. **B.** Quantification of cells with activated caspase-3/7 after treatment with CDDO or CDDO-Me or up to 24 hours. Data represent the mean ± SD of four independent experiments; $*P < 0.05$ and $**P < 0.01$ *vs.* Ctrl.

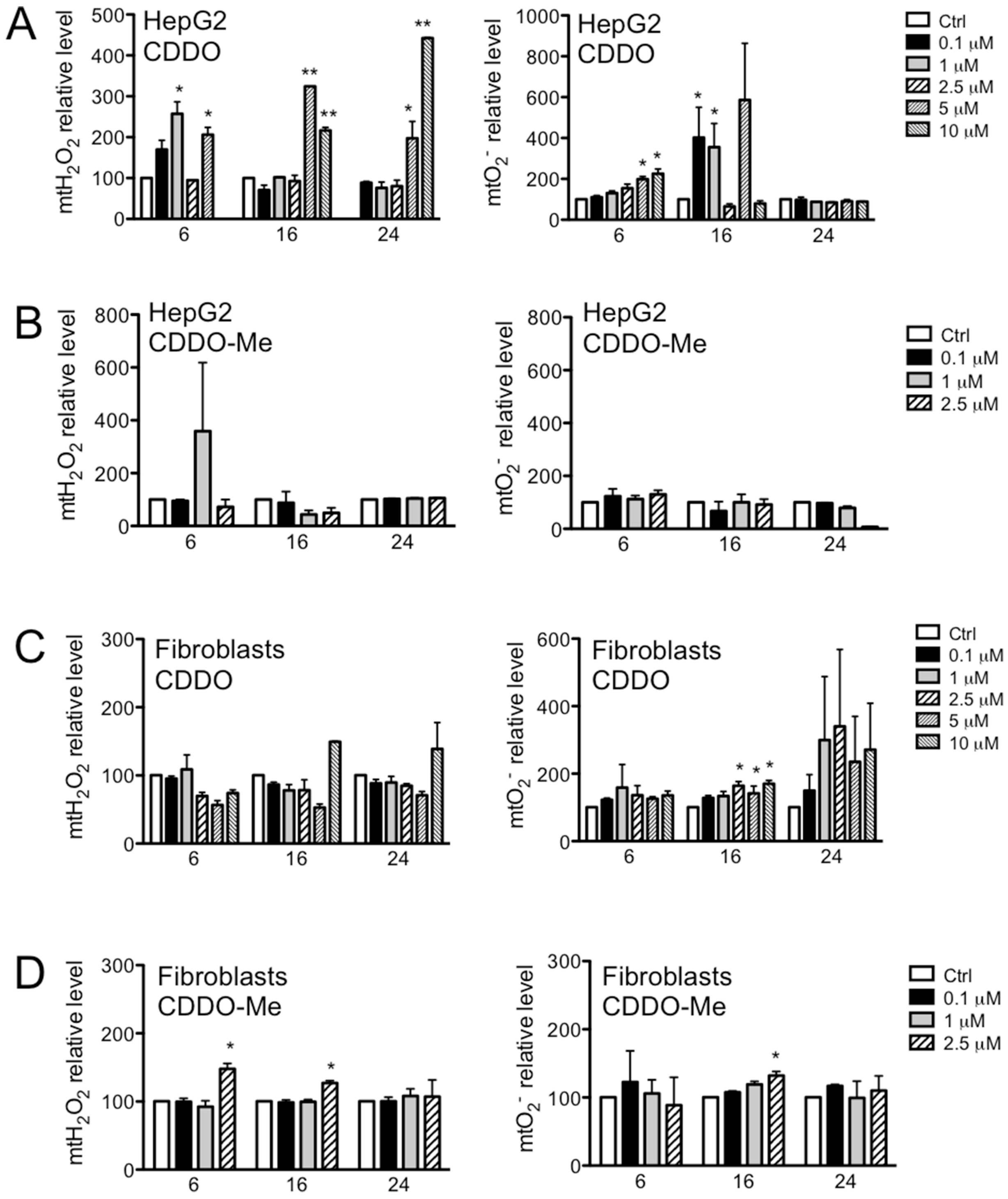

Supplementary Figure S4: CDDO and CDDO-Me increase the levels of mitochondrial reactive oxygen species in HepG2 cells but not in primary fibroblasts. A. Quantification of mitochondrial hydrogen peroxide (mtH_2O_2) and mitochondrial anion superoxide (mtO_2^-.) in HepG2 cells treated with CDDO for up to 24 hours. Data are expressed as percentage of increase in median fluorescence intensity (MFI) and represent the mean ± SD of three independent experiments; $*P < 0.05$ and $**P < 0.01$ *vs.* Ctrl. **B.** Quantification of mtH_2O_2 and mtO_2^{-} in HepG2 cells treated with CDDO-Me for 6, 16 and 24 hours. Data are expressed as percentage of increase in MFI and represent the mean ± SD of three independent experiments. **C.** Quantification of mtH_2O_2 and mtO_2^{-} in primary cultures of fibroblasts treated with CDDO for up to 24 hours. Data are expressed as percentage of increase in MFI and represent the mean ± SD of three independent experiments; $*P < 0.05$ *vs.* Ctrl. **D.** Quantification of mtH_2O_2 and mtO_2^{-} in primary cultures of fibroblasts treated with CDDO-Me for 6, 16 and 24 hours. Data are expressed as percentage of increase in MFI and represent the mean ± SD of three independent experiments.

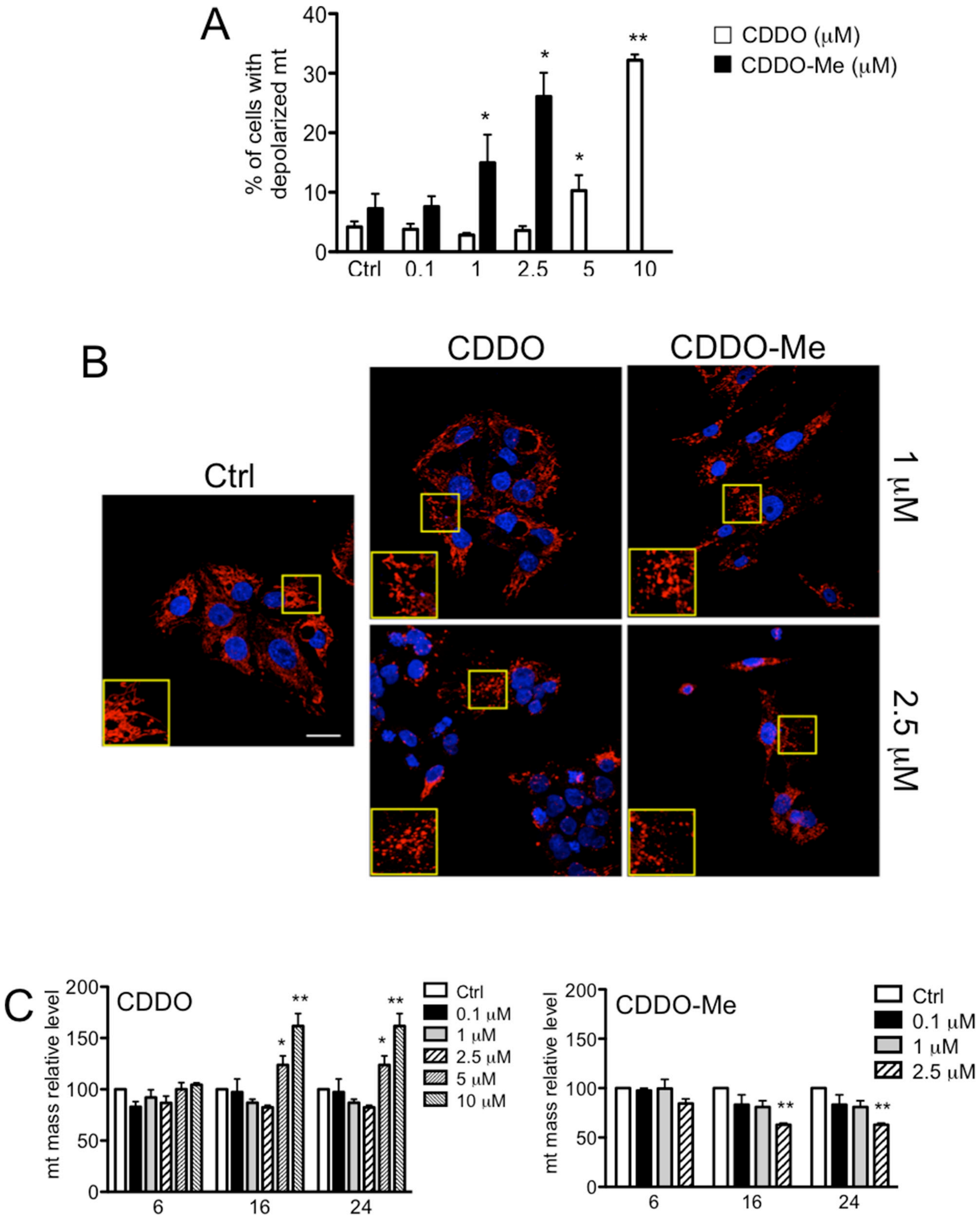

Supplementary Figure S5: CDDO and CDDO-Me depolarize mitochondria and alter mitochondrial morphology in HepG2 cells. A. Quantification of cells with depolarized mitochondria. Values represent the mean ± SD of three independent experiments, $*P < 0.05$ and $**P < 0.01$ *vs*. Ctrl. **B.** Representative confocal microscopy images showing mitochondria in HepG2 cells treated with CDDO and CDDO-Me. Red fluorescence represents mitochondria labelled with anti-human mitochondrial protein and with goat anti-rabbit $F(ab')_2$ Alexa 647. Nuclei were counterstained with DAPI. **C.** Detection of mitochondrial mass in HepG2 cells treated with DMSO (Ctrl) and increasing concentrations of CDDO and CDDO-Me for up to 24 hours, as revealed by flow cytometry. Data are expressed as percentage of increase in MFI in comparison to Ctrl, set to 100%, and represent the mean ± SD of four independent experiments; $*P < 0.05$ and $**P < 0.01$ *vs*. Ctrl.

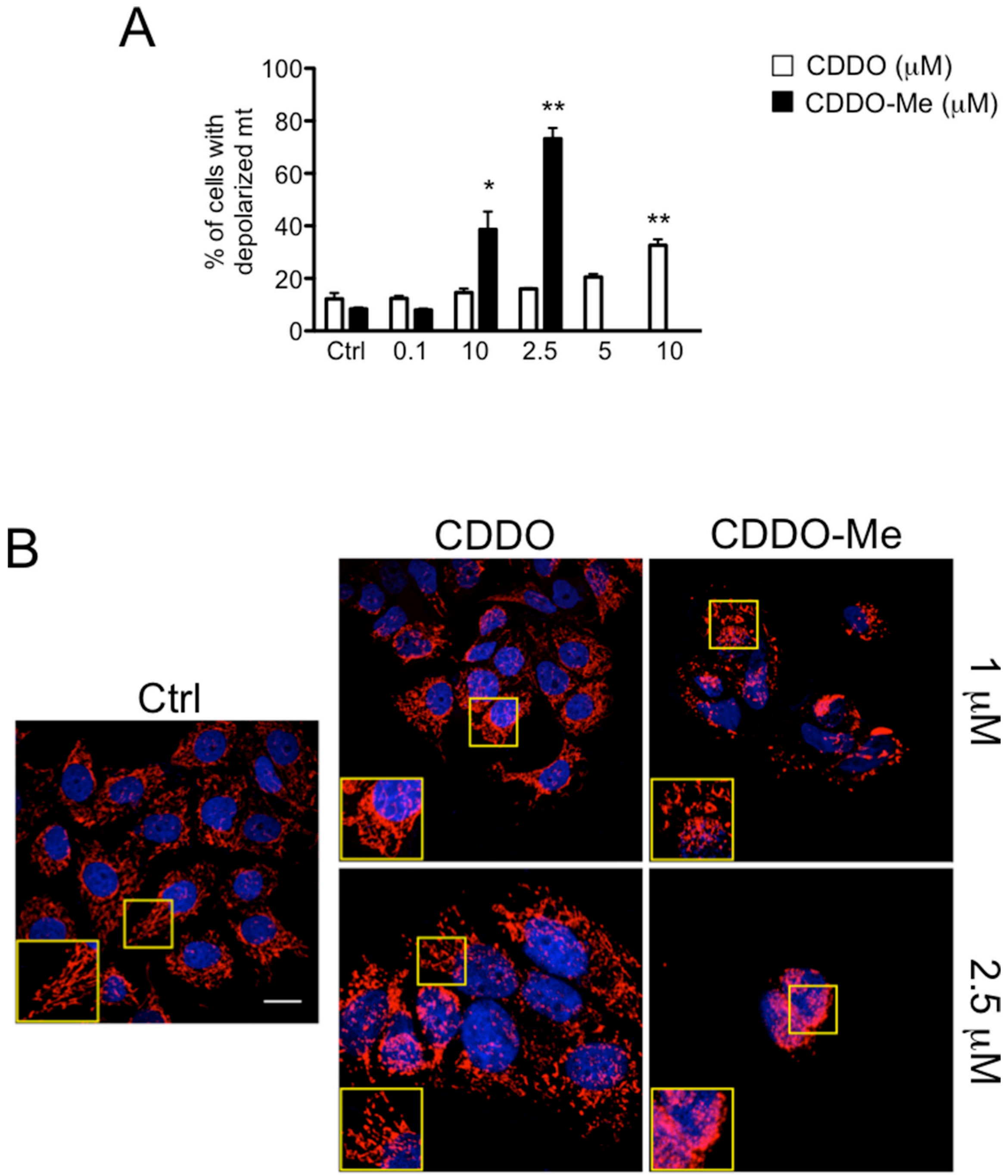

Supplementary Figure S6: CDDO and CDDO-Me depolarize mitochondria and alter mitochondrial morphology in MCF7 cells. A. Quantification of cells with depolarized mitochondria. Values represent the mean ± SD of three independent experiments, *P < 0.05 and **P < 0.01 *vs*. Ctrl. **B.** Representative confocal microscopy images showing mitochondria in MCF7 cells treated with CDDO and CDDO-Me. Red fluorescence represents mitochondria labelled with anti-human mitochondrial protein and with goat anti-rabbit F(ab')$_2$ Alexa 647. Nuclei were counterstained with DAPI.

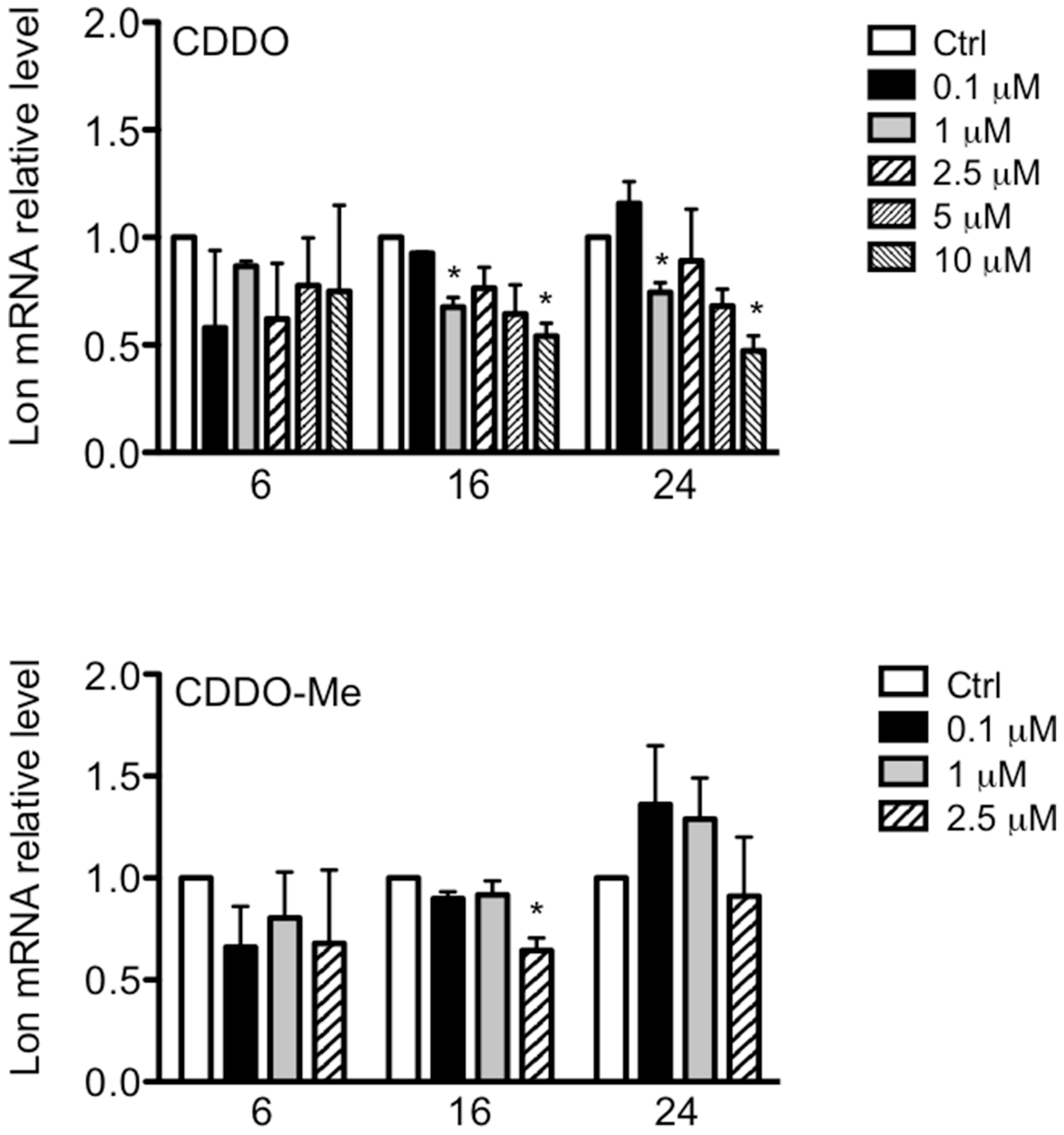

Supplementary Figure S7: CDDO and CDDO slightly modulate mRNA levels of Lon. Quantification of Lon mRNA in RKO cells treated with CDDO and CDDO-Me for up to 24 hours, as revealed by real time PCR. Data are expressed as the mean ± SD of four independent experiments; *$P < 0.05$ *vs*. Ctrl.
